# Selenium suppressed the LPS-induced oxidative stress of bovine endometrial stromal cells through Nrf2 pathway with high cortisol background

**DOI:** 10.1093/jas/skae260

**Published:** 2024-09-02

**Authors:** Luying Cui, Fangling Zheng, Min Zhang, Zhihao Wang, Xia Meng, Junsheng Dong, Kangjun Liu, Long Guo, Heng Wang, Jianji Li

**Affiliations:** College of Veterinary Medicine, Yangzhou University, Jiangsu Co-innovation Center for Prevention and Control of Important Animal Infectious Disease and Zoonoses, Yangzhou, Jiangsu, PR China; Joint International Research Laboratory of Agriculture and Agriproduct Safety of the Ministry of Education, Yangzhou, Jiangsu, PR China; International Research Laboratory of Prevention and Control of Important Animal Infectious Diseases and Zoonotic Diseases of Jiangsu Higher Education Institutions, Yangzhou University, Yangzhou, Jiangsu, PR China; College of Veterinary Medicine, Yangzhou University, Jiangsu Co-innovation Center for Prevention and Control of Important Animal Infectious Disease and Zoonoses, Yangzhou, Jiangsu, PR China; Joint International Research Laboratory of Agriculture and Agriproduct Safety of the Ministry of Education, Yangzhou, Jiangsu, PR China; International Research Laboratory of Prevention and Control of Important Animal Infectious Diseases and Zoonotic Diseases of Jiangsu Higher Education Institutions, Yangzhou University, Yangzhou, Jiangsu, PR China; College of Veterinary Medicine, Yangzhou University, Jiangsu Co-innovation Center for Prevention and Control of Important Animal Infectious Disease and Zoonoses, Yangzhou, Jiangsu, PR China; Joint International Research Laboratory of Agriculture and Agriproduct Safety of the Ministry of Education, Yangzhou, Jiangsu, PR China; International Research Laboratory of Prevention and Control of Important Animal Infectious Diseases and Zoonotic Diseases of Jiangsu Higher Education Institutions, Yangzhou University, Yangzhou, Jiangsu, PR China; College of Veterinary Medicine, Yangzhou University, Jiangsu Co-innovation Center for Prevention and Control of Important Animal Infectious Disease and Zoonoses, Yangzhou, Jiangsu, PR China; Joint International Research Laboratory of Agriculture and Agriproduct Safety of the Ministry of Education, Yangzhou, Jiangsu, PR China; International Research Laboratory of Prevention and Control of Important Animal Infectious Diseases and Zoonotic Diseases of Jiangsu Higher Education Institutions, Yangzhou University, Yangzhou, Jiangsu, PR China; College of Veterinary Medicine, Yangzhou University, Jiangsu Co-innovation Center for Prevention and Control of Important Animal Infectious Disease and Zoonoses, Yangzhou, Jiangsu, PR China; Joint International Research Laboratory of Agriculture and Agriproduct Safety of the Ministry of Education, Yangzhou, Jiangsu, PR China; International Research Laboratory of Prevention and Control of Important Animal Infectious Diseases and Zoonotic Diseases of Jiangsu Higher Education Institutions, Yangzhou University, Yangzhou, Jiangsu, PR China; College of Veterinary Medicine, Yangzhou University, Jiangsu Co-innovation Center for Prevention and Control of Important Animal Infectious Disease and Zoonoses, Yangzhou, Jiangsu, PR China; Joint International Research Laboratory of Agriculture and Agriproduct Safety of the Ministry of Education, Yangzhou, Jiangsu, PR China; International Research Laboratory of Prevention and Control of Important Animal Infectious Diseases and Zoonotic Diseases of Jiangsu Higher Education Institutions, Yangzhou University, Yangzhou, Jiangsu, PR China; College of Veterinary Medicine, Yangzhou University, Jiangsu Co-innovation Center for Prevention and Control of Important Animal Infectious Disease and Zoonoses, Yangzhou, Jiangsu, PR China; Joint International Research Laboratory of Agriculture and Agriproduct Safety of the Ministry of Education, Yangzhou, Jiangsu, PR China; International Research Laboratory of Prevention and Control of Important Animal Infectious Diseases and Zoonotic Diseases of Jiangsu Higher Education Institutions, Yangzhou University, Yangzhou, Jiangsu, PR China; College of Veterinary Medicine, Yangzhou University, Jiangsu Co-innovation Center for Prevention and Control of Important Animal Infectious Disease and Zoonoses, Yangzhou, Jiangsu, PR China; Joint International Research Laboratory of Agriculture and Agriproduct Safety of the Ministry of Education, Yangzhou, Jiangsu, PR China; International Research Laboratory of Prevention and Control of Important Animal Infectious Diseases and Zoonotic Diseases of Jiangsu Higher Education Institutions, Yangzhou University, Yangzhou, Jiangsu, PR China; College of Veterinary Medicine, Yangzhou University, Jiangsu Co-innovation Center for Prevention and Control of Important Animal Infectious Disease and Zoonoses, Yangzhou, Jiangsu, PR China; Joint International Research Laboratory of Agriculture and Agriproduct Safety of the Ministry of Education, Yangzhou, Jiangsu, PR China; International Research Laboratory of Prevention and Control of Important Animal Infectious Diseases and Zoonotic Diseases of Jiangsu Higher Education Institutions, Yangzhou University, Yangzhou, Jiangsu, PR China; College of Veterinary Medicine, Yangzhou University, Jiangsu Co-innovation Center for Prevention and Control of Important Animal Infectious Disease and Zoonoses, Yangzhou, Jiangsu, PR China; Joint International Research Laboratory of Agriculture and Agriproduct Safety of the Ministry of Education, Yangzhou, Jiangsu, PR China; International Research Laboratory of Prevention and Control of Important Animal Infectious Diseases and Zoonotic Diseases of Jiangsu Higher Education Institutions, Yangzhou University, Yangzhou, Jiangsu, PR China

**Keywords:** bovine endometrial stromal cells, oxidative stress, cortisol, Na_2_SeO_3_, Nrf2

## Abstract

Stress and infection seriously threaten the reproductive performance and health of dairy cows. Various perinatal stresses increase plasma cortisol concentrations in cows, and chronically high cortisol levels may increase the incidence and severity of the uterine diseases. Selenium (**Se**) enhances antioxidant capacity of cows. The aim of this study was to explore how Se affects the oxidative stress of primary bovine endometrial stromal cells (**BESC**) with high cortisol background. The levels of reactive oxygen species **(ROS)** and other biomarkers of oxidative stress were measured using flow cytometry and assay kits. The changes in nuclear NF-E2-related factor 2 **(Nrf2)** pathway were detected by Western blot, qPCR, and immunofluorescence. The result showed that lipopolysaccharide **(LPS)** increased (*P* < 0.01) ROS and malondialdehyde **(MDA)** content and reduced (*P* < 0.01) superoxide dismutase **(SOD)** concentration, provoking BESC oxidative stress. The elevated levels of cortisol resulted in the accumulation (*P* < 0.05) of ROS and MDA and inhibition (*P* < 0.05) of SOD in unstimulated BESC but demonstrated an antioxidative effect in LPS-stimulated cells. Pretreatment with Se reduced (*P* < 0.01) the levels of ROS and MDA, while increasing (*P* < 0.05) the antioxidant capacities and the relative abundance of gene transcripts and proteins related to the Nrf2 pathway in BESC. This antioxidant effect was more pronounced in the presence of high cortisol level. In conclusion, cortisol alone induced the oxidative damage but provided an antioxidant protection in the presence of LPS. Se alleviated the LPS-induced cellular oxidative stress, which is probably achieved through activating Nrf2 pathway. At high cortisol levels, Se supplement has a more significant protective effect on BESC oxidative stress. This study provided evidence for the protective role of Se in bovine endometrial oxidative damage of stressed animals and suggested the potential regulatory mechanism in vitro.

## Teaser

This study helps reveal the regulatory mechanism of Se on endometrial oxidative stress under the condition of high cortisol.

## Graphical Abstract

**Graphical Abstract:**
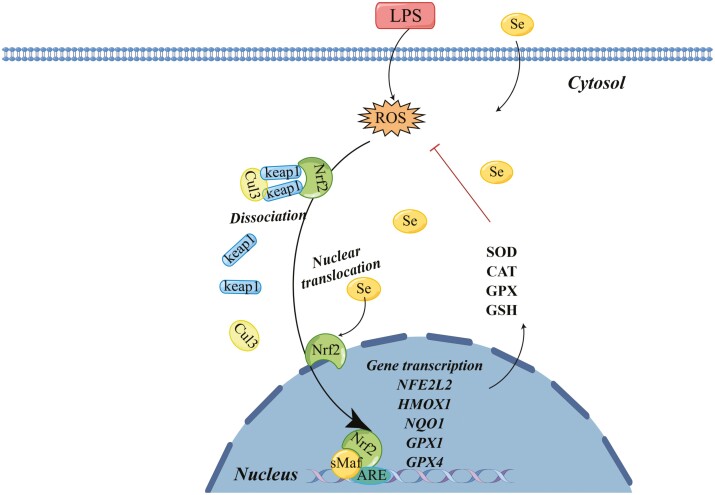

## Introduction

After calving, the bovine uterine lumen is almost always contaminated with a wide range of bacteria, which can invade the endometrium and cause diseases such as metritis and endometritis. The bovine uterine infections can affect up to 40% of dairy cows and negatively impact their reproductive performance, resulting in huge economic losses (Sheldon et al., 2009). *Escherichia coli* infection in the uterus during the early postpartum period contributes to the disease process (Çömlekcioğlu et al., 2024). The *E. coli* virulence factor lipopolysaccharide (**LPS**) has been proven to provoke inflammation and damage in bovine endometrial cells (Adeniran et al., 2022; Cui et al., 2023a).

Stress is a risk factor for the bovine uterine infections (Barragan et al., 2018). During the transitional period, cows experience various stressors, which increase the plasma cortisol level (Lucy, 2019). Cortisol is a common indicator of stress in animals. An elevated cortisol caused by acute stressors may evoke an immuno-enhancing effect and regulate glucolipid metabolism (Carroll and Forsberg, 2007; Kuo et al., 2015). Normally in healthy cows, the cortisol level was about 6 ng/mL before calving, with a sharp rise to 19.2 ng/mL on parturition day, and dropped to 6 ng/mL two days postpartum (Hudson et al., 1976). Some studies have reported a significantly higher cortisol concentration in cows developing fetal membrane retention (Pathak et al., 2015). An early research indicated that cows with puerperal complications, including purulent vaginal discharge, retained placenta, dystocia, milk fever, and vaginal lacerations, had significantly higher basal cortisol levels, and had higher occurrence of uterine bacterial infection during the second week when compared with the healthy cows (Torres et al., 1997).

Oxidative stress is associated with the increased incidence and severity of uterine infections and is one of the key components of this pathological process (Wrzecińska et al., 2021). Oxidative stress occurs when the excessive formation of reactive oxygen species (**ROS**) cannot be scavenged by the antioxidant system of living organisms (Apel and Hirt, 2004). On one hand, the periparturient cows experience a negative energy balance, which can lead to an overproduction of lipid peroxides and ROS (Celi and Gabai, 2015). On the other hand, *E. coli* can cause oxidative lowering of antioxidant enzymes and lipid peroxidation in endometrial tissue and cells (Jiang et al., 2021; Cui et al., 2023a). The decreased expression of antioxidant genes has been reported in the endometrium of cows with endometritis (Shaukat et al., 2024).

Selenium (Se) is an essential trace mineral of fundamental importance and is closely related to bovine health and production performance. For dairy cows, the Se requirement is 300 μg/kg DM per day (Mehdi and Dufrasne, 2016). Serum Se concentration with a range from 70 to 79 μg/L is considered adequate in dairy cows (Gong and Xiao, 2015). Se deficiency is associated with oxidative stress, and a sufficient supply of Se facilitates antioxidation in dairy cows. Se supplementation has been shown to reduce the incidence of metritis, ovarian cysts, retained placenta, and mastitis (Xiao et al., 2021). Gong and Xiao (2018) suggested that the metabolic activity increased during the perinatal period, and supplementation of Se slightly above the recommended requirements may be beneficial to alleviate the oxidative stress of dairy cows. Hall et al. (2014) also found that feeding Se-yeast supplement to Se-replete cows during late gestation improved their antioxidant status and immune responses during early lactation.

Se supplement can improve bovine resistance to uterine infection during early lactation and can relieve the LPS-induced oxidative stress and inflammatory response in bovine endometrial cells (Adeniran et al., 2022; Cui et al., 2023b). The endometrial epithelium is sloughed during the postpartum period, which allows microbes to reach the stroma (Chapwanya et al., 2009). The effect and mechanism of Se on the oxidative stress of bovine endometrial stromal cells (**BESC**) remains unclear. Moreover, the effect of Se on oxidative stress of BESC at high cortisol background has not been reported.

The aim of this study was to explore how Se affects the oxidative stress of BESC with high cortisol background. In this research, we detected the oxidative stress biomarkers, the antioxidant capacity, and the changes in Nrf2 pathway at gene and protein levels. The results showed that Se supplement alleviated the oxidative stress, increased the antioxidant capacity, and activated the Nrf2 pathway of BESC stimulated with LPS. Cortisol alone led to an increase in intracellular ROS, but inhibited the LPS-induced ROS accumulation. Se still played a protective role against LPS-induced oxidative stress in BESC in the presence of high cortisol level.

## Materials and Methods

### Animal ethics

All experimental procedures were approved by the Animal Care and Use Committee of Yangzhou University (202103317).

### Cell culture

The bovine uteri were collected at a local abattoir from postpubertal non-pregnant Holstein dairy cows with hoof diseases. These cows showed no evidence of genital diseases or microbial infections based on the presence of foul smell, characteristic visual appearance, and vaginal discharge. The uteri were kept on ice until further processing in the laboratory. The stage of the reproductive cycle was determined by examination of ovarian morphology and vasculature, and the uteri at ovarian stage I (days 1 to 4 of the estrous cycle) were selected for endometrial cell culture because peripheral plasma progesterone concentrations are basal, similar to those of postpartum cows (Turner et al., 2014).

The uterine surface was washed with iodophor and 75% alcohol, and flushed clean with sterile saline containing 1,000 U/mL penicillin/streptomycin. A stab incision was made in the center of the horn, just above the intercornual ligament, and then the uterine horn was cut open longitudinally. The endometrial secretions were cleansed using a 75% alcohol-soaked cotton ball and the epithelium was removed by scraping with a sterile blade. Then the intercaruncular endometrial stripes were dissected from the myometrial layer with surgical scissors and forceps. The stripes were chopped into small pieces. After repeated rinses with phosphate-buffered saline (**PBS**) supplemented with 200 U/mL penicillin/streptomycin, the minced tissue was digested in DMEM/F-12 (D8900, Sigma, MO, USA) containing 0.25% collagenase II (C6885-5G, Sigma). After 1 h incubation in a shaking water bath at 37 °C, the cell suspension was filtered through a 40-μm mesh to remove the undigested material, and the filtrate was washed three times by centrifugation (153 × *g* for 5 min) with PBS containing 200 U/mL penicillin/streptomycin. The cells were resuspended in DMEM/F-12 containing 15% fetal bovine serum and 100 IU/mL of penicillin, 100 μg/mL of streptomycin, 0.25 μg/mL of amphotericin B (BL142A, Biosharp, Beijing, China). The cells were cultured in 75 cm^2^ flasks (707001, NEST, Wuxi, China), and the medium was changed after 12 h to facilitate the selective attachment of stromal cells and the removal of epithelial cells. The cells were incubated in a humidified atmosphere with 5% CO_2_ at 37 °C, and the cell culture medium was changed every 24 to 48 h. When the cell confluence reached approximately 90%, the cells were subcultured. The cells were digested at 37 °C using 0.25% trypsin (0458-50g, Livning, Beijing, China) for 40 to 60 s, and the digestion was terminated using the complete medium. At this time, most stromal cells can fall off from the cell culture bottle wall after blowing, while the epithelial cells are still firmly attached to the wall. The purity of stromal cell population was determined to be more than 95% by the detection of vimentin using immunocytochemistry (Supplementary material 1).

### Experiment design and treatments

LPS (L2880, *E. coli* O55:B5), cortisol (H0888), and sodium selenite (S5261) were purchased from Sigma-Aldrich (MO, USA). These powders were dissolved in DMEM/F-12, then filtered and diluted to the concentrations of 1 mg/mL, 1 mM, and 1 μg/mL for LPS, sodium selenite (Na_2_SeO_3_), and cortisol, respectively, and were stored at −20 °C.

About 1 μg/mL LPS was used to induce the oxidative stress of BESC. Selection of the Na_2_SeO_3_ concentration in the range of 1 to 16 μM was based on previous studies (Cui et al., 2023b). The physiological level of cortisol in cattle ranges from 5 to 30 ng/mL. Based on a preliminary study in our laboratory (Dong et al., 2018), a concentration of 30 ng/mL was selected as the high cortisol level.

In experiment 1, we explored the effect of cortisol on the oxidative stress biomarkers, the antioxidant capacities, and the Nrf2 pathway in BESC with or without LPS stimulation. First, the time-course changes (0 to 12 h) in the levels of ROS and malondialdehyde (**MDA**), the superoxide dismutase (**SOD**) concentration, and the relative abundance of mRNA transcripts and proteins of the Nrf2 pathway (*NEF2L2*, NFE2 like bZIP transcription factor 2; Nrf2, nuclear factor erythroid 2-related factor 2; HO1/*HMOX1*, heme oxygenase 1; NOQ1, NAD(P)H quinone dehydrogenase 1) were observed in BESC stimulated with LPS. Then, the cells were treated with cortisol of various concentrations (0, 5, 15, 30, 300 ng/mL) with or without the presence of LPS, to observe the effect of cortisol on the biomarkers of oxidative stress, the antioxidant enzyme concentration (SOD; GPX, glutathione peroxidase; CAT, catalase; GSH, glutathione), the total antioxidant capacity (T-AOC), and the Nrf2 pathway. The groups were as follows: the control group, the LPS treatment group, the LPS and cortisol co-treatment groups.

In experiment 2, we observed the effect of Se on the oxidative stress of LPS-stimulated BESC with high cortisol background. We determined the safe concentrations of Na_2_SeO_3_ for BESC by using a CCK8 assay and selected 4 μM Na_2_SeO_3_ for the follow-up experiment. The concentration of 30 ng/mL was regarded as the high cortisol level. After Se pretreatment for 12 h, the cells were challenged with LPS and cortisol for 12 h. The groups were as follows: the control group, the LPS group, the LPS and Na_2_SeO_3_ co-treatment group (LPS-Se group), the LPS, cortisol and Se co-treatment group (LPS-COR-Se group).

### Cell viability and cytotoxicity assays

The Cell Counting Kit-8 (A311-02-AA, Vazyme, Nanjing, China) was used to evaluate the impact of Se, LPS either alone or in combination, on the cell viability of BESC. The cells were seeded into wells of a 96-well plate (2 × 10^3^ cells per well) and grown to 80% confluence. The medium was replaced with DMEM/F-12 containing LPS and/or Se. After 24 h treatment, the Cell Counting Kit-8 solution was added to each well, followed by an additional incubation for 2 h. The optical density was read at 450 nm using a microplate reader (Tecan, Austria).

The lactate dehydrogenase (**LDH**) concentration in cell culture medium was detected using an LDH assay kit (A020-2-2, Jiancheng Bioengineering Institute, Nanjing, China) to determine cytotoxicity of Na_2_SeO_3_. The cells were seeded into 6-well plates at a density of 1 × 10^6^ cells per well and grown to 80% confluence at 37 °C with 5% CO_2_. The cells of Se supplement groups were treated with DMEM/F-12 containing LPS and/or Se (1 to 4 μM) for 24 h. Then the cell culture medium was collected. According to the instructions of the kit, the absorbance value was read at the wavelength of 450 nm with a microplate reader.

### Detection of intracellular ROS

The BESC was seeded into 6-well plates at a density of 1 × 10^6^ cells per well. The cells were treated according to the group design for 12 h. Then the cells were stained using a cell ROS assay kit (S0033S, Beyotime, Shanghai, China), and the content of ROS was analyzed by a FACScan flow cytometer (Becton Dickinson). After being digested with trypsin with ethylenediaminetetraacetic acid for 2 min, the digestion was terminated with DMEM/F-12 containing 15% fetal bovine serum. The cell suspension was centrifuged at 153 × *g* for 5 min, and the supernatant was discarded. The cells were collected and suspended in serum-free DMEM/F-12 containing DCFH-DA (DCFH-DA:DMEM/F-12 = 1:1000), and were incubated in a cell incubator at 37 °C for 30 min and mixed upside down every 2 to 3 min. The cells were then washed 3 times with serum-free DMEM/F-12. The ROS level was detected by flow cytometry at a 488-nm excitation wavelength.

### Detection of SOD, CAT, GPX, GSH, T-AOC, and MDA

The BESC was seeded into 6-well plates at a density of 1 × 10^6^ cells per well. At the end of the treatment, the cells were collected and dissolved in 200 μL PBS. The mixture was sonicated every 5 s at four intervals on ice and then centrifuged at 1,530 × *g* for 10 min to obtain the cell-free supernatants. A bicinchoninic acid protein assay kit (P0010, Beyotime) was used to determine the protein concentration in the supernatant. The concentrations of CAT (A007-1-1), SOD (A001-3-2), GSH (A006-2-1), and GPX (A005-1-2) and the levels of total antioxidant capacity (T-AOC) (A015-2-1) and MDA (A003-4-1) were detected by the commercial kits purchased from Jiancheng Bioengineering Institute.

### RNA extraction and quantitative PCR

The cells were plated in 6-well plates (1 × 10^6^ cells per well) and grown to 80% fusion. After the treatment as previously described, the cells were washed with PBS, and the total RNA was subsequently extracted using a Trizol reagent (DP424, TIANGEN, Beijing, China) according to the manufacturer’s protocol. The extracted RNA was quantified using a Nanodrop 2000 spectrophotometer (Thermo, USA). The absorption ratio (A260/280) was determined to be between 1.8 and 2.0. About 1 μg of DNase-1-digested total RNA was reverse transcribed into cDNA using 4 μL 5 × *TransScript* Uni All-in-One SuperMix (AU341, TransGen, Beijing, China) and 1 μL gDNA Remover (AU341, TransGen). The quantitative PCR was carried out using a CFX 96 Real-Time PCR Detection System (Bio-Rad, USA). The cDNA was amplified in a 20-μL reaction solution containing 2 μL of cDNA template, 10 μL of 2 × ChamQ SYBR qPCR Master Mix (Q311-02/03, Vazyme), 1 pM sense and antisense gene-specific primers, and double distilled water. The amplification cycle program was 95 °C, 30 s; 95 °C, 10 s, 60 °C, 30 s, for 40 cycles; 95 °C, 15 s; melt curve 60 to 95 °C with increment of 0.5 °C.

The primers were designed based on the sequence of *Beta-actin* (*ACTB*)*, NFE2L2, HMOX1,* and *NOQ1* genes of *Bos taurus* published in GenBank, using Primer 6.0 and synthesized by Beijing Tsingke Biotech Co., Ltd. The primer sequences were shown in Table 1. *ACTB* was used as an internal control, the CT values of *ACTB* unchanged regardless of the treatment, and the results were calculated using the 2^−ΔΔct^ method.

**Table 1.** The list of primer sequences

Target gene	Primer sequences (5ʹ-3ʹ)	Size	Accession number
*ACTB*	F: CATCACCATCGGCAATGAGC	156 bp	NM-173979.3
	R: AGCACCGTGTTGGCGTAGAG		
*NFE2L2*	F: CCCAGTCTTCACTGCTCCTC	165 bp	NM-001011678.2
	R: TCAGCCAGCTTGTCATTTTG		
*HMOX1*	F: GGCAGCAAGGTGCAAGA	221 bp	NM-001014912.1
	R: GAAGGAAGCCAGCCAAGAG		
*NQO1*	F: AACCAACAGACCAGCCAATC	154 bp	NM-001034535.1
	R: CACAGTGACCTCCCATCCTT		

### Western blot analysis

The cells were plated in 6-well plates (1 × 10^6^ cells per well) and grown to 80% fusion. After the treatment as previously described, the cells were washed with PBS to extract the total protein. The cells were scraped and dissolved into radioimmunoprecipitation assay lysate containing protease inhibitors and protein phosphatase inhibitors. The mixture was split on ice for 15 min and shaken at high speed every 5 min, then centrifuged at 1,530 × *g* at 4 °C for 10 min. The cells were plated in 100 mm cell-culture dishes (1 × 10^6^ cells per well) and grown to 80% fusion. After the treatment as previously described, the cell nuclear protein was extracted according to the instructions of the Nuclear and Cytoplasmic Protein Extraction Kit (P0028, Beyotime). The protein concentration of the supernatant was measured using the bicinchoninic acid protein assay kit (P0010, Beyotime). After the protein supernatant was mixed with the SDS-PAGE loading buffer (P1015, Solarbio, Beijing, China), the mixture was denatured by heating in a metal bath at 100 °C for 10 min. After cooling to room temperature and centrifugation at 1,530 × *g* for 5 min, the supernatant was taken for electrophoresis. The proteins were separated by 10% sodium dodecyl sulfate–polyacrylamide gel electrophoresis and transferred to polyvinylidene fluoride (**PVDF**) membranes. The PVDF membranes were incubated with 5% skimmed milk diluted by Tris Buffered Saline with Tween-20 (TBST, 0.05% Tween-20 Tris-HCl buffer) at room temperature for 2 h, and then incubated with primary antibodies including Nrf2 (66504-1-Ig, Proteintech, Chicago, IL, USA), HO1 (AF5393, Affinity Biosciences, Changzhou, China), NQO1 (DF6437, Affinity Biosciences), glucocorticoid receptor (**GR**, WL02695, Wanleibio, Shenyang, China), Lamin B1 (12987-1-AP, Proteintech) and beta-actin (AF7018, Affinity Biosciences) at 4 °C overnight. The membranes subjected to Nrf2, HO1, NQO1, GR, beta-actin, and Lamin B1 were incubated with HRP-conjugated goat anti-rabbit secondary antibody (7074, Cell Signaling Technology, MA, USA) at room temperature for 1.5 h. These primary antibodies of Nrf2 (dilution ratio 1:1,000), GR (dilution ratio 1:500), Lamin B1 (dilution ratio 1:5,000), and beta-actin (dilution ratio 1:10,000) were diluted with SuperKine Enhanced Antibody Dilution Buffer (BMU103-CN, Abbkine, Wuhan, China). The secondary antibody was diluted by TBST containing 5% skimmed milk. The bands were detected using the chemiluminescence assay. The gray value of the protein bands was quantified by the Image J software (National Institutes of Health, ML, USA), and was then normalized to Beta-actin or Lamin B1.

### Immunofluorescence staining

The cells were seeded in 24-well plates containing glass slides. After the treatment as previously described, the cells were fixed with 4% paraformaldehyde (BL539A, Biosharp) for 15 min, and 0.4% Triton × 100 (ST797, Beyotime) was used to penetrate the cell membrane at 37 °C for 15 min. After blockage with 5% bovine serum albumin (A8020, Solarbio) at 37 °C for 1.5 h, the cells were incubated with Nrf2 antibody (dilution ratio 1:200) at 4 °C overnight. The slides were subsequently washed with PBS, and were incubated with the FITC-conjugated secondary antibody (A0423, Beyotime) at 37 °C for 1.5 h in the dark. The nuclei were stained with 4, 6-diamidino-2-phenylindole (C1005, Beyotime) at 37 °C for 15 min in the dark. The distribution of Nrf2 protein was observed by a laser confocal microscope (Leica TCS SP8, Leica Corp, Germany). The mean fluorescence intensity of Nrf2 in the nucleus was quantified and analyzed by the Image J software.

### Statistical analysis

Each experiment was repeated at least three times. The experimental data were analyzed by one-way ANOVA using SPSS 21.0 software (IBM, NY, USA), followed by the least significant difference test. Data were presented as the means ± SEM. The *P* value of less than 0.05 indicated a significant difference between groups.

## Results

### LPS-induced oxidative stress in BESC

**Figure 1.**
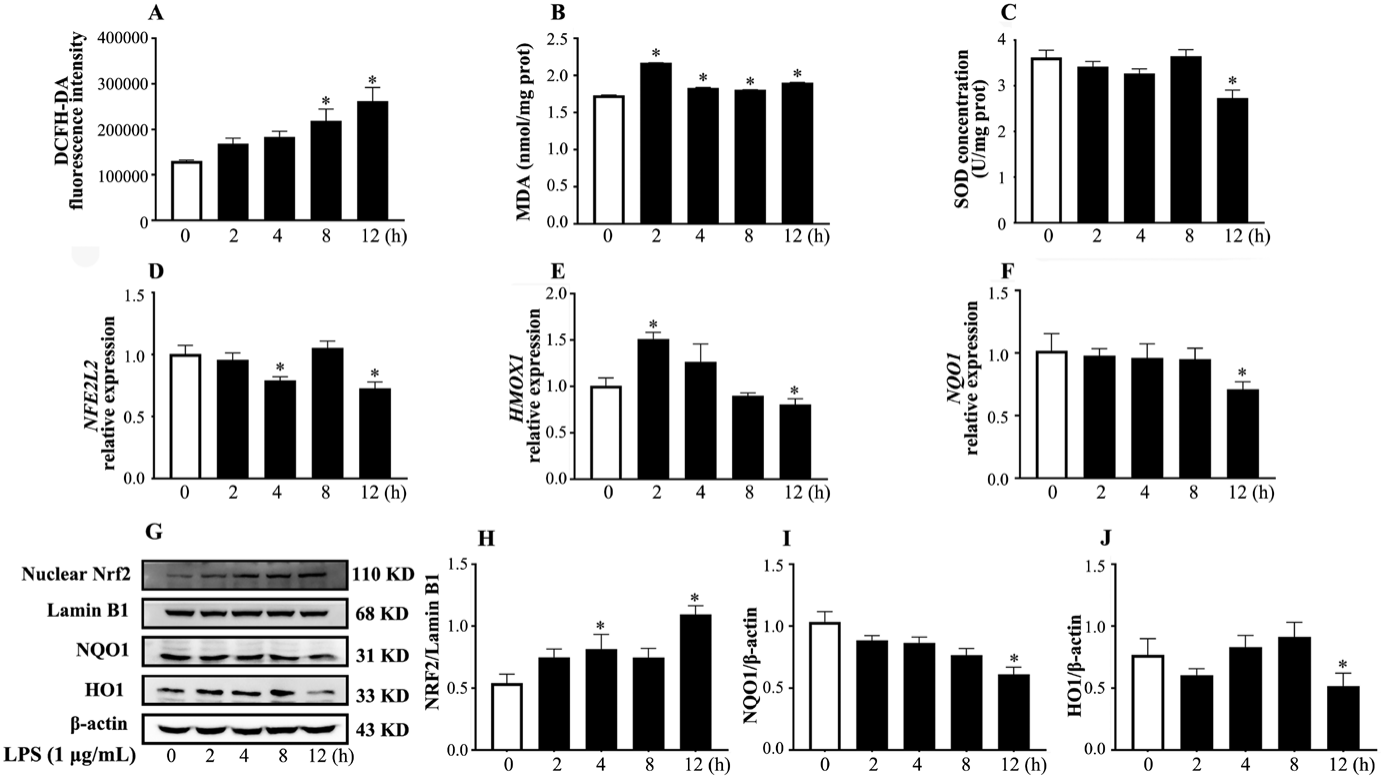
The time-course changes in the oxidative stress biomarkers (A to C) and the relative abundance of mRNA transcripts and proteins related to the Nrf2 pathway (D to J) in primary BESC stimulated with 1 µg/mL lipopolysaccharide. The protein blots were quantified by densitometry and the densitometry data for HO1 and NQO1 were normalized by β-actin, nuclear Nrf2 was normalized by Lamin B1. ROS, reactive oxygen species; MDA, malondialdehyde; SOD, superoxide dismutase; *NFE2L2*, NFE2 like bZIP transcription factor 2; Nrf2, nuclear factor erythroid 2-related factor 2; HO1/*HMOX1*, heme oxygenase 1; *NOQ1*, NAD(P)H quinone dehydrogenase 1. The data were presented as the means ± SEM (n = 3). *P < 0.05 and **P < 0.01 vs. the control group.

We first determined whether LPS can induce an oxidative stress of BESC. As shown in Figure 1, LPS treatment for 12 h caused increased (*P* < 0.05) contents of ROS and MDA, and a reduced (*P* < 0.05) SOD concentration. In addition, there was a decrease (*P* < 0.05) in the relative abundance of mRNA transcripts for *NFE2L2, HMOX1*, and *NOQ1*, and the corresponding protein level of HO1 and NQO1 in LPS group at 12 h. Meanwhile, the protein level of nuclear Nrf2 increased (*P* < 0.05). These results suggested that 1 μg/mL LPS treatment provoked BESC oxidative stress.

### Cortisol exhibited distinct effects on BESC oxidative status in the presence or absence of LPS

**Figure 2.**
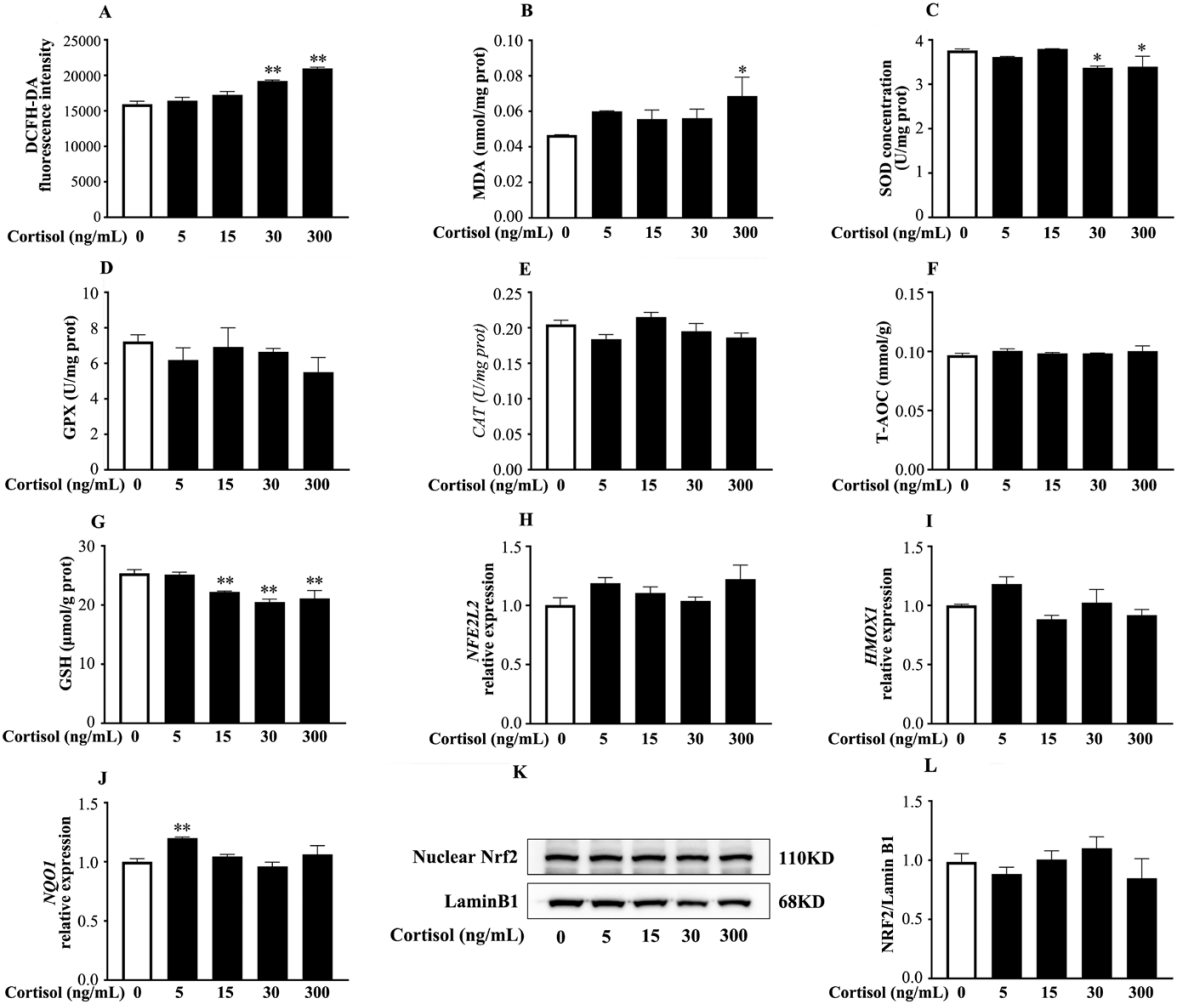
High-level cortisol treatment alone induced oxidative stress without affecting the Nrf2 pathway in primary BESC. The cells were treated with cortisol (5, 15, 30, 300 µg/mL) for 12 h, followed by the detection of ROS (A), MDA (B), the antioxidant biomarkers (C to G), and the relative abundance of mRNA transcripts and proteins of Nrf2 pathway (H to L). The nuclear protein blots were quantified by densitometry and the densitometry data for Nrf2 was normalized by Lamin B1. ROS, reactive oxygen species; MDA, malondialdehyde; SOD, superoxide dismutase; CAT, catalase; GPX, glutathione peroxidase; T-AOC, total antioxidant capacity; GSH, glutathione; *NFE2L2*, NFE2 like bZIP transcription factor 2; Nrf2, nuclear factor erythroid 2-related factor 2; HO1/*HMOX1*, heme oxygenase 1; *NOQ1*, NAD(P)H quinone dehydrogenase 1. The data were presented as the means ± SEM (n = 3). *P < 0.05 and **P < 0.01 vs. the control group.

In BESC with quiescent state (Figure 2), cortisol caused no change at low concentrations (5 and 15 ng/mL), but generally resulted in an increased (*P* < 0.05) ROS and MDA level, and a decreased (*P* < 0.05) protein contents of SOD and GSH at higher concentrations (30 and 300 ng/mL). Cortisol from 0 to 300 ng/mL showed no effect (*P* > 0.05) on GPX, CAT, and T-AOC. In addition, we observed no variation (*P* > 0.05) in the relative abundance of Nrf2-related mRNA transcripts and nuclear Nrf2 protein in BESC treated with cortisol. These results suggested that high cortisol level (30 and 300 ng/mL) caused oxidative stress, but did not influence Nrf2 pathway in BESC.

**Figure 3.**
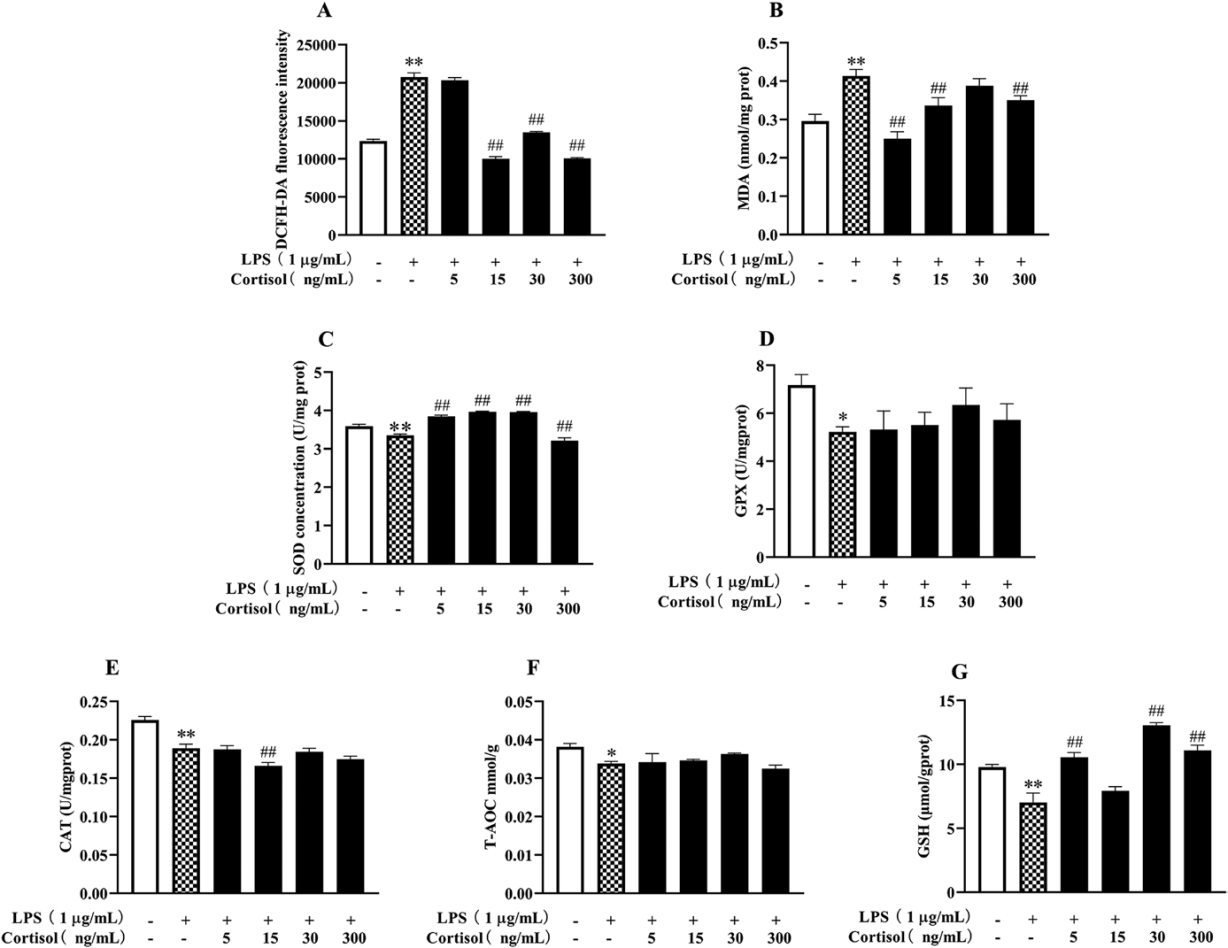
High-level cortisol treatment presented an antioxidative effect in primary BESC stimulated with lipopolysaccharide (LPS). The cells were treated with LPS and cortisol (5, 15, 30, 300 µg/mL) for 12 h. The changes in biomarkers of oxidative stress, including ROS (A), MDA (B), SOD (C), GPX (D), CAT (E), T-AOC (F), and GSH (G) were determined. ROS, reactive oxygen species; MDA, malondialdehyde; SOD, superoxide dismutase; GPX, glutathione peroxidase; CAT, catalase; T-AOC, total antioxidant capacity; GSH, glutathione. The data were presented as the means ± SEM (n = 3). *P < 0.05 and **P < 0.01 vs. the control group. ^#^*P* < 0.05 and ^##^*P* < 0.01 vs. the LPS group.

In BESC with LPS stimulation (Figure 3), the increased (*P* < 0.05) ROS and MDA level, and the decreased SOD (*P* < 0.05) concentration were consistent with the aforementioned results in Figure 1. Moreover, LPS reduced (*P* < 0.05) the protein contents of CAT and GPX and the levels of T-AOC and GSH.

**Figure 4.**
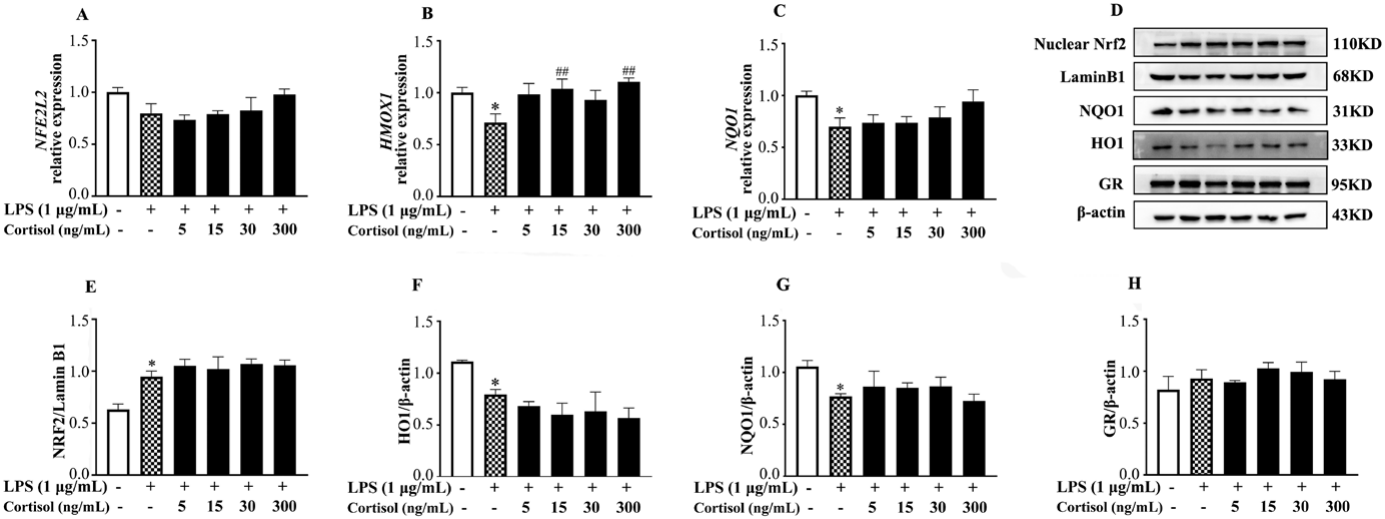
The effect of high-level cortisol on the Nrf2 pathway in primary BESC stimulated with lipopolysaccharide (LPS). The cells were treated with LPS and cortisol (5, 15, 30, 300 µg/mL) for 12 h. The relative abundance of mRNA transcripts for *NFE2L2* (A), *HMOX1* (B), and *NQO1* (C), and the protein abundance of nuclear Nrf2 (E), total HO1 (F) and NQO1 (G) were detected by qPCR and Western blot, respectively. Moreover, the protein abundance of GR was detected (H). The protein blots were quantified by densitometry and the densitometry data for nuclear Nrf2 was normalized by LaminB1, and the HO1, NQO1, and GR were normalized by β-actin. *NFE2L2*, NFE2 like bZIP transcription factor 2; Nrf2, nuclear factor erythroid 2-related factor 2; HO1/*HMOX1*, heme oxygenase 1; *NOQ1*, NAD(P)H quinone dehydrogenase 1; GR, glucocorticoid receptor. The data were presented as the means ± SEM (n = 3). *P < 0.05 and **P < 0.01 vs. the control group. ^#^*P* < 0.05 and ^##^*P* < 0.01 vs. the LPS group.

Compared with the LPS group, cortisol generally resulted in a reduction (*P* < 0.05) in ROS and MDA levels, and an increase in (*P* < 0.05) the GSH concentration in BESC. Except for the elevated (*P* < 0.05) SOD concentration, the addition of cortisol generally showed no effect (*P* > 0.05) on the levels of GPX, CAT, and T-AOC in BESC stimulated with LPS. As shown in Figure 4, compared with the LPS group, there was a greater (*P* < 0.05) abundance of *HMOX1* transcript in the cells treated with 15 and 300 ng/mL cortisol and LPS. The changes in *NFE2L2* and *NQO1* transcripts were similar to that of *HMOX1* but without statistical significance (*P* > 0.05). Analogously, no difference (*P* > 0.05) was found in the protein levels of HO1, NQO1, and nuclear Nrf2 between the LPS group and the co-treatment groups of LPS and cortisol (*P* > 0.05). There was no change (*P* > 0.05) in GR protein abundance throughout the experiment.

### Na_2_SeO_3_ exerts antioxidant effects under high cortisol background

**Figure 5.**
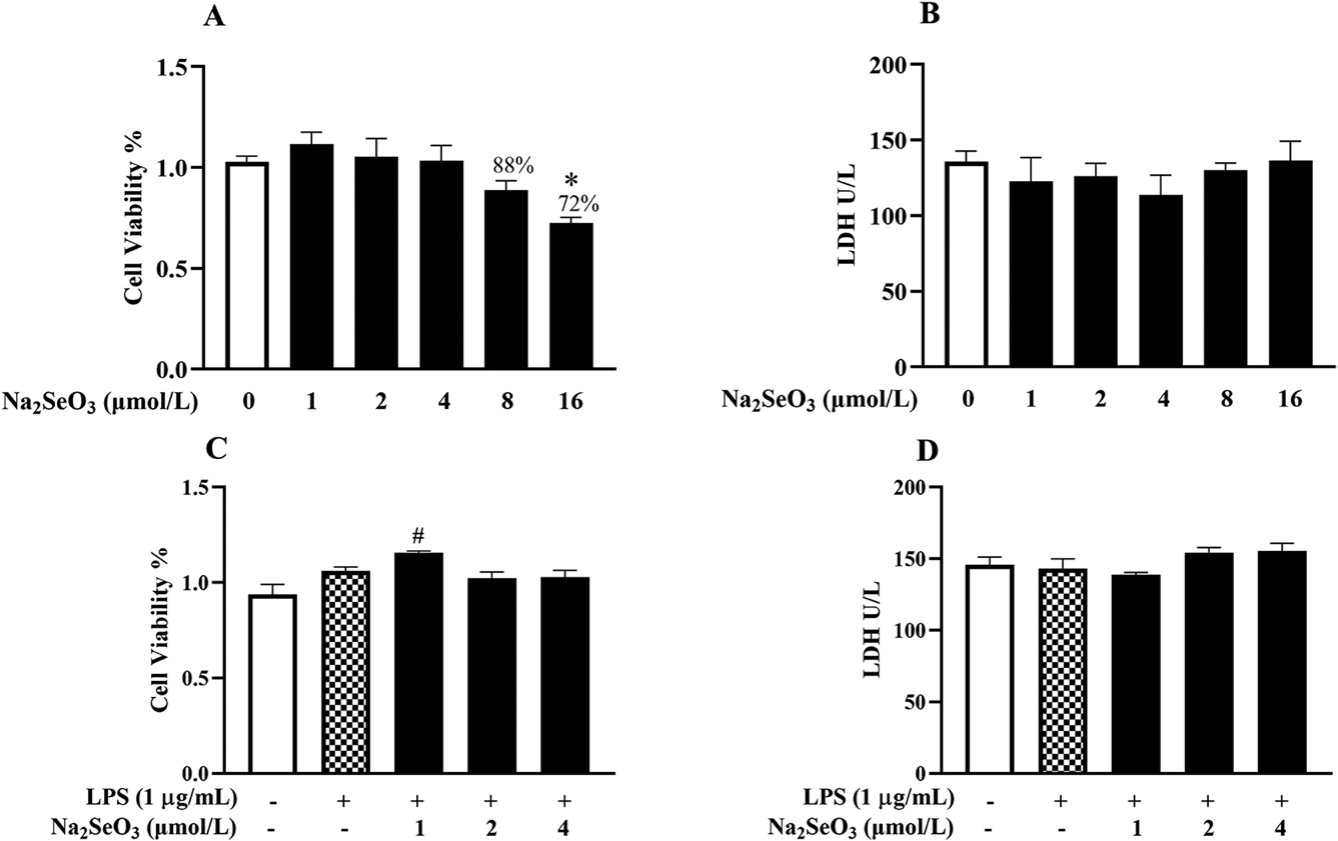
The effect of different concentrations of Na_2_SeO_3_ on the viability (A, C) and the LDH release (B, D) of primary BESC. The cells were treated with Na_2_SeO_3_, or co-treated with Na_2_SeO_3_ and lipopolysaccharide (LPS, 1 μg/mL) for 24 h. LDH, lactate dehydrogenase. The data were presented as means ± SEM (n = 6). *P < 0.05 and **P < 0.01 vs. the blank group; ^#^*P* < 0.05 and ^##^*P* < 0.01 vs. the LPS group.

We first observed the effect of Na_2_SeO_3_ on cell viability and LDH release with or without LPS. As shown in Figure 5, Na_2_SeO_3_ did not affect BESC viability at concentrations from 1 to 8 μM. Exposure of cells to 1 to 4 μM Na_2_SeO_3_ and LPS did not cause LDH leakage or cell number reduction. Therefore, we selected 4 μM Na_2_SeO_3_ for the subsequent experiment.

**Figure 6.**
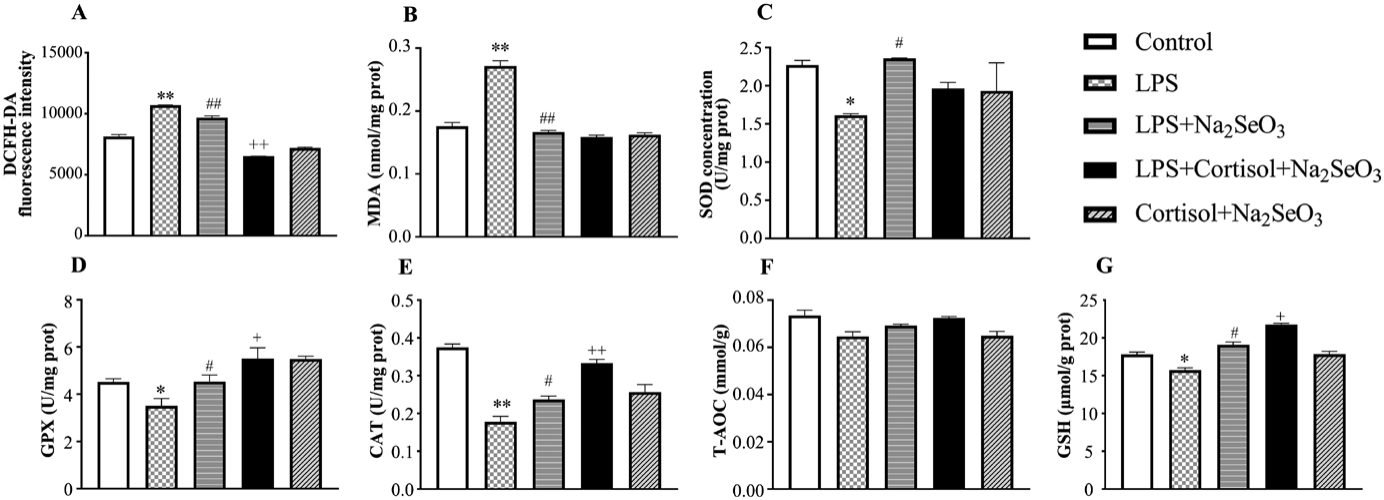
The effect of Se on the oxidative status of primary BESC with high cortisol background. After Se pretreatment for 12 h, the cells were co-treated with 1 μg/mL lipopolysaccharide (LPS), 30 ng/mL cortisol and/or 4 μM Na_2_SeO_3_ for 12 h. The changes in ROS (A), MDA (B), and the antioxidant biomarkers (C-G) were detected. ROS, reactive oxygen species. MDA, malondialdehyde; SOD, superoxide dismutase; GPX, glutathione peroxidase; CAT, catalase; T-AOC, total antioxidant capacity; GSH, glutathione. The data were presented as the means ± SEM (n = 3). *P < 0.05 and **P < 0.01 vs. the control group. ^#^*P* < 0.05 and ^##^*P* < 0.01 vs. the LPS group. ^+^*P* < 0.05 and ^++^*P* < 0.01 vs. the LPS-Se group.

In the presence of LPS (Figure 6), Se pretreatment reduced (*P* < 0.05) the levels of ROS and MDA, and increased (*P* < 0.05) the concentrations of SOD, GPX, CAT, and GSH, but not T-AOC (*P* > 0.05). Compared with LPS-Se group, the addition of cortisol further reduced (*P* < 0.05) ROS release and MDA content and increased (*P* < 0.05) the concentrations of CAT, GSH, and GPX, but not (*P* > 0.05) SOD and T-AOC.

**Figure 7.**
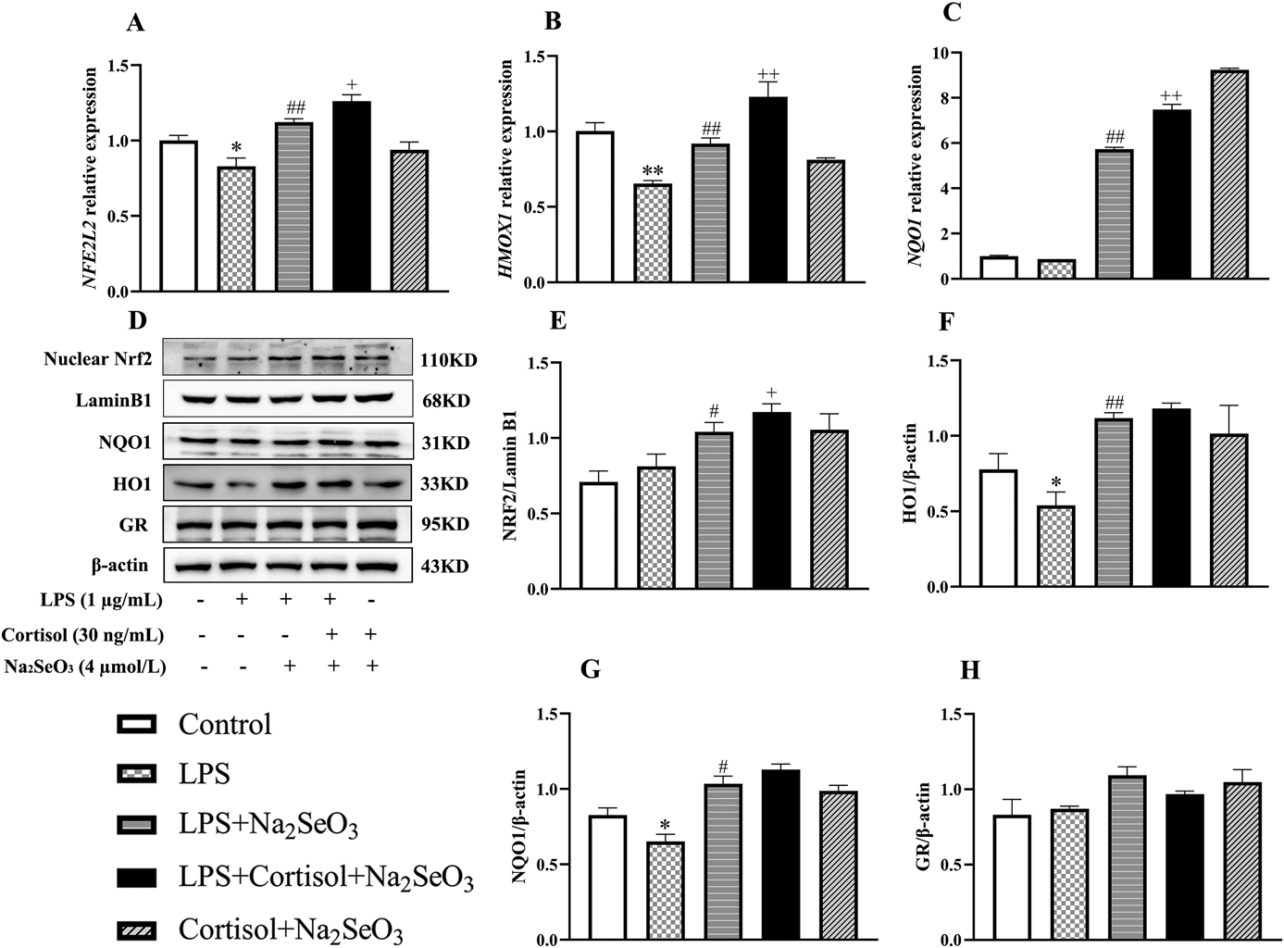
The effect of Se on the Nrf2 pathway of primary BESC with high cortisol background. After Se pretreatment for 12 h, the cells were co-treated with 1 μg/mL lipopolysaccharide (LPS), 30 ng/mL cortisol and/or 4 μM Na_2_SeO_3_ for 12 h The relative abundance of mRNA transcripts for *NFE2L2* (A), *HMOX1* (B), and *NQO1* (C), and the protein abundance of nuclear Nrf2 (E), total HO1 (F) and NQO1 (G) were detected by qPCR and Western blot, respectively. The protein abundance of GR was detected (H). The protein blots were quantified by densitometry and the corresponding data were normalized by LaminB1 or β-actin. *NFE2L2*, NFE2 like bZIP transcription factor 2; Nrf2, nuclear factor erythroid 2-related factor 2; HO1/*HMOX1*, heme oxygenase 1; *NOQ1*, NAD(P)H quinone dehydrogenase 1; GR, glucocorticoid receptor. The data were presented as the means ± SEM (n = 3). *P < 0.05 and **P < 0.01 vs. the control group. ^#^*P* < 0.05 and ^##^*P* < 0.01 vs. the LPS group. ^+^*P* < 0.05 and ^++^*P* < 0.01 vs. the LPS-Se group.

As depicted in Figure 7, compared with the LPS group, Se caused an increase (*P* < 0.05) in the relative abundance of Nrf2 pathway-related mRNA transcripts and proteins in BESC of LPS-Se group. Compared with the LPS-Se group, there was a greater abundance (*P* < 0.05) in the mRNA transcripts for *NEF2L2*, *HMOX1*, and *NQO1*, and the nuclear Nrf2 protein in BESC of LPS-COR-Se group. The protein abundance of HO1 and NQO1 tended to be greater in cells of LPS-COR-Se group than those of LPS-Se group (*P* = 0.052 and *P* = 0.051, respectively). The GR protein level did not change (*P* > 0.05) among the treatment groups.

**Figure 8.**
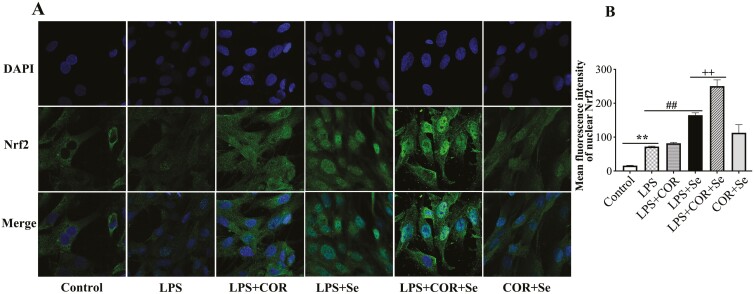
The effect of Se on the Nrf2 translocation in primary BESC with high cortisol background. After Se pretreatment for 12 h, the cells were co-treated with 1 μg/mL lipopolysaccharide (LPS), 30 ng/mL cortisol and/or 4 μM Na_2_SeO_3_ for 12 h. The translocation of Nrf2 protein from the cytoplasm into the nucleus was visualized (A) and quantified (B). Scale bar represented 10 μm. The data were presented as the means ± SEM (n = 3). *P < 0.05 and **P < 0.01 vs. the control group. ^#^*P* < 0.05 and ^##^*P* < 0.01 vs. the LPS group. ^+^*P* < 0.05 and ^++^*P* < 0.01 vs. the LPS-Se group.

The effect of Se on Nrf2 distribution with high cortisol background was detected using immunofluorescence staining (Figure 8). In the control group, the fluorescence signal of Nrf2 was only distributed in the cytoplasm, and there was almost no fluorescence signal in the nucleus. LPS-stimulated (*P* < 0.05) the nuclear translocation of Nrf2 in BESC, and the green fluorescence signal was evenly distributed in cytoplasm and nucleus. In comparison with the LPS group, the mean nuclear green fluorescence intensity unchanged in the LPS-COR group, but was promoted (*P* < 0.05) in the LPS-Se group. The level of Nrf2 in the nucleus was the higher (*P* < 0.01) in the LPS-COR-Se group compared to the LPS-Se or LPS-COR group.

## Discussion

### LPS caused BESC oxidative stress with Nrf2 pathway inhibition

To maintain a physiological low-level ROS, the body is equipped with a robust antioxidant defense system made up of the endogenous non-enzymatic antioxidants, such as bilirubin and GSH, the enzymatic antioxidants, including SOD, CAT, and GPX, and the exogenous antioxidants (Ayemele et al., 2021). SOD protects cells against oxidative stress by controlling the superoxide concentration. CAT efficiently dismutates hydrogen peroxide to H_2_O and O_2_. GPX facilitates the decomposition of hydrogen peroxide and the reduction process of reduced glutathione to oxidized glutathione (Jomova et al., 2024). Oxidative stress occurs when excessive ROS production or defects in the antioxidant defense system occur. ROS is able to oxidatively modify a variety of substances, and MDA is the byproduct and an indicator of lipid peroxidation. The *E. coli* LPS has been proven to cause the overproduction of ROS and MDA (Adeniran et al., 2022), and has been found to decrease CAT and SOD activities in bovine endometrial cells (Gugliandolo et al., 2020; Fu et al., 2021a). Previously, we have induced the oxidative stress of primary bovine endometrial epithelial cells (**BEEC**) by 1μg/mL LPS (Cui et al., 2023a). When applying the same LPS dosage to BESC this time, we observed similar results, including the increased ROS and MDA contents, and the decreased antioxidant capacity (SOD, GPX, and CAT), suggesting BESC oxidative stress.

Nrf2 pathway is the principal protective response to oxidative stress. Under homeostatic conditions, Nrf2 binds to Kelch-like ECH-associated protein 1 to form a complex and is degraded through the ubiquitin proteasome pathway. After stimulation with electrophilic reagents, there is a conformational change of the complex that prevents Nrf2 release and inhibits Nrf2 ubiquitination. This enables the newly translated Nrf2 to bypass the complex and undergo nuclear translocation. It binds to small Maf proteins and antioxidant response element (**ARE**), and regulates the downstream gene transcription, such as NQO1, HO1, thioredoxins, glutathione reductase, and SOD (Egbujor et al., 2023). HO1 degrades heme and generates the antioxidant molecules, including biliverdin and CO, to alleviate cellular oxidative stress and inflammation. NQO1 prevents ROS generation by reducing electrophilic quinones to hydroquinone, and mediates the regeneration of vitamin E after free radical attack (Ross and Siegel, 2021). LPS has been reported to inhibit Nrf2 phosphorylation and the protein expression of HO1 and NQO1 in AR42J cells (Lee et al., 2022). Interestingly, regarding the effect of LPS on the Nrf2 pathway in BEEC, opposite results have been reported by Fu et al. In one study, they treated BEEC with 1 μg/mL LPS for 3 h, and found a decrease in *HMOX1* and *NQO1* expression without detecting nuclear Nrf2 (Fu et al., 2023). In another study, they found an increase in the gene expression of *NFE2L2*, *HMOX1*, and *NQO1*, and the protein level of nuclear Nrf2 in BEEC treated with 10 μg/mL LPS for 24 h (Fu et al., 2021b). These reports suggested that the changes in the key proteins and genes of Nrf2 pathway are related to the concentration and treatment time of LPS. According to our result, 1 μg/mL LPS stimulation for 12 h resulted in the decreased abundance of *NFE2L2*, *HMOX1*, and *NQO1* transcripts, and the HO1 and NQO1 proteins, but an increased protein level of nuclear Nrf2. The *NFE2L2* gene encodes Nrf2 protein. Although unable to provide a definitive explanation for the inconsistent results between the nuclear Nrf2 level and the relative abundance of *NFE2L2* transcript, we supposed it may be related to the fact that the temporal and spatial discordance between mRNA and protein expression is more profound under non-steady-state conditions (Fan et al., 2024). Based on the fact that the changes in oxidative stress markers were in accordance with the change in the mRNA transcripts of genes downstream of Nrf2 pathway, we proposed that the elevated nuclear Nrf2 level did not reflect the transient cell status. A thorough observation of the time-course change in nuclear Nrf2 level and ARE activity in BESC may help to reveal the underlying mechanism.

### The specific impact of cortisol on BESC oxidative status with or without LPS is independent of the Nrf2 pathway

An acute exposure to cortisol and norepinephrine has been reported to cause ROS/RNS release and DNA damage in breast cancer cell lines MDA-MB-231 and MCF-7 through GR-mediated signaling (Flaherty et al., 2017). Other studies have also shown that glucocorticoids (**GC**) can increase ROS accumulation in neuronal culture (You et al., 2009; Bose et al., 2010). Similar to these studies, we observed that cortisol of 30 and 300 ng/mL increased the ROS level and decreased the GSH and SOD concentrations in BESC. Mitochondria is a major source of ROS, and Du et al. (2009) confirmed that the long-term high-dose cortisol led to mitochondrial oxidative enhancement and promoted mitochondrial ROS production. However, in the previous study, we observed a distinct result in BEEC, that the levels of ROS and MDA reduced after cortisol (5, 15, and 30 ng/mL) treatment alone (data unpublished, Supplementary Material 2). The physiological and pharmacological actions of GC are mediated by the GR (Mao et al., 2023). The 11β-hydroxysteroid dehydrogenase (11βHSD) is involved in the regulation of cortisol metabolism and is also the ligand for GR (Chapman et al., 2013). The different effects of cortisol on ROS between BESC and BEEC may be associated with the different 11βHSD subtypes, in which 11βHSD-1 was localized in epithelial cells, and 11βHSD-2 was localized in endometrial stromal cells in rats (Burton et al., 1998). 11βHSD-2 is an NAD-dependent high affinity 11β-dehydrogenase that inactivates GC (Chapman et al., 2013). This may also be the reason why we did not observe changes in GR protein abundance in BESC after cortisol treatment. How GR modulates BESC oxidative status, and whether the 11βHSD subtype is involved in the process deserves further investigation. In addition, we examined the effect of cortisol on GSH, and the results showed that intracellular GSH concentration was reduced by cortisol, so did the report from von Mässenhausen et al. (2022), that dexamethasone treatment reduced GSH in HT1080 cells. According to our result, the GPX concentration tended to decrease after cortisol treatment alone, this observation was in line with the reports that chronic GC consumption alters the gene expression of selenoprotein P and iodothyronine deiodinase 2 (Rock and Moos, 2009; Wray et al., 2019), and that GC decreased GPX1 protein expression and activity in hippocampal cells (You et al., 2009).

The effect of GC on Nrf2 activity is expected to be highly tissue and cell specific. A recent study claiming that nuclear localization of Nrf2 was not affected by dexamethasone, but dexamethasone treatment increased GR recruitment to ARE thus negatively affecting the binding of Nrf2 to ARE in HepG2 cells (Alam et al., 2017). Another study suggested that GC inhibited Nrf2 activation and protein expression through GR in HEK-293 cells and hepatic H4IIE cells (Kratschmar et al., 2012). In BESC treated with cortisol, we observed no change in Nrf2 pathway, probably suggesting that cortisol modulates BESC oxidative status without a direct action on Nrf2 pathway. A growing body of evidence suggests that GR also act via non-genomic mechanisms to elicit rapid cellular responses that do not require changes in gene expression (Samarasinghe et al., 2012). These signaling mechanisms involve phosphatidylinositol-3-kinase (**PI3K**), protein kinase C (**PKC**), and mitogen-activated protein kinases pathway (Oakley and Cidlowski, 2013). Whether and how these mechanisms play a role in the regulation of oxidative status by cortisol in BESC remains to be investigated.

In the presence of LPS, we observed that cortisol resulted in the decreased ROS and MDA levels, and increased SOD and GSH concentrations, indicating that cortisol alleviated oxidative damage and improved antioxidant capacity of BESC with oxidative stress. This was contrary but not contradictory to the effect of cortisol alone because GC suppresses inflammation. Cortisol has been proven to inhibit LPS-induced inflammation in bovine endometrial epithelial and stromal cells (Dong et al., 2018; Fang et al., 2022). The decreased release of pro-inflammatory mediators can be linked to the reduced oxidative intermediates. Moreover, in BEEC, we observed similar results that cortisol relieved the LPS-induced formation of ROS and MDA and inhibition of antioxidant enzyme activity (data unpublished, Supplementary Material 3). Therefore, the antioxidative effect of cortisol, in the presence of LPS, is probably related to its anti-inflammation properties.

### The enhanced antioxidative effect of Se in the presence of cortisol is related to the Nrf2 pathway

In practice, Se supplement for dairy cows can minimize the harmful effects of excessive free radicals, improving the health status and reducing the disease incidence in perinatal period (Abuelo et al., 2015). Se status in the sampled dairy cows was assessed using the following scoring scheme: deficiency (<40 µg/L), marginal status (50 to 70 µg/L), sufficient supply (70 to 90 µg/L), and abundant supply (>100 µg/L) (Pavlata et al., 2000). The plasma Se level of toxicity has not been reported in clinic. According to a study, Se-yeast supplement to cows during late gestation has been found to improve the postpartum antioxidant capacity without negative effects, and the whole blood Se concentration reached 371 ± 7 µg/L (Hall et al., 2014). In the present study, we have determined the Se concentration in the basal medium being 45.63 μg/L. The final concentration of Se after the addition of 4 μM Na_2_SeO_3_ was 315.84 μg/L. Se supplementation has been proven to promote the vitality and antioxidant capacity of various cell types (Sun et al., 2020; Adeniran et al., 2022). For example, in bovine mammary epithelial cells, Se treatment reduced the intracellular ROS and MDA level, and improved the SOD, CAT, and GPX activities (Sun et al., 2020). This is consistent with our observations. Then, we investigated the role of the Nrf2 signaling pathway as an antioxidant mechanism of Se and observed that Se increased the relative abundance of *NFE2L2* and *NQO1* transcripts and the corresponding proteins, and promoted the nuclear translocation of Nrf2. Similarly, it has been demonstrated that Se promoted the expression of Nrf2 transcription factor-related genes at both mRNA and protein levels in a bovine endometrial cell line during LPS stimulation (Adeniran et al., 2022). Se functions as a gatekeeper of cellular redox homeostasis in the form of selenoproteins (Labunskyy et al., 2014). We detected that Se supplementation resulted in a greater abundance of mRNA transcripts for *GPX1* and *GPX4* in BESC with or without the presence of LPS (data unpublished, Supplementary Material 4). Therefore, we speculated that the addition of Se activated the Nrf2 pathway, increased antioxidant enzyme activities, and reduced ROS and MDA accumulation, thereby alleviating LPS-induced oxidative stress of BESC. The regulatory effect of Se on oxidative stress may be related to GPX. In subsequent studies, Nrf2 inhibitor ML385 or silencing/overexpression techniques will be applied to confirm the downstream regulation of Nrf2 and the antioxidant effects of GPX or other selenoproteins in BESC.

When cortisol was combined with Se to treat BESC and observe their impact on LPS-induced oxidative stress, we found that the ROS release was reduced and the activities of CAT and GPX were increased compared to the cells in LPS-Se group. One explanation for this enhanced antioxidation of BESC in LPS-COR-Se group could be the anti-inflammatory effect of cortisol because the reduced release of proinflammatory cytokines and mediators by cortisol is associated with a reduced ROS accumulation. Previously, we have demonstrated that, in epithelial cells, Se supplementation with high cortisol background attenuated LPS-induced proinflammatory gene expression and had a stronger anti-inflammatory effect than Se alone (Cui et al., 2023b). In addition, Se supplementation showed a pronounced antioxidant effect in the context of high cortisol in BEEC (data unpublished, Supplementary Material 5). In the current result, the greater abundance of *NFE2L2* and *NOQ1* transcripts in LPS-COR-Se co-treated cells, and the obvious green fluorescence in the nucleus indicated a further activation of Nrf2. Although there was no change with statistical significance, the protein levels of HO1 and NQO1 tended to be higher in BESC of the LPS-COR-Se group than those of the LPS-Se group. This tendency was in accordance with the changes in *NFE2L2, HMOX1,* and *NQO1* transcripts, and the nuclear Nrf2 translocation. Cortisol has been shown to activate the extranuclear signaling molecule PIK3 in BEEC (Dong et al., 2019). It is possible that the enhanced antioxidant effect of Se in the context of high cortisol is related to the activation of PI3K (Oakley and Cidlowski, 2013), which promotes Nrf2 phosphorylation, stability, and the subsequent transcriptional activity (He et al., 2020). In BESC, whether cortisol regulates Nrf2 activity through this mechanism needs to be further verified.

Se and cortisol may have mutual influence in various aspects. On one hand, numerous in vivo studies have demonstrated that vitamin E and Se administration has the potential to decrease cortisol concentration in many stress conditions, such as transport (Jung et al., 2023), pregnancy (Dimri et al., 2010), and surgery (Mudron and Rehage, 2018). Sodium selenite supplementation has been found to relieve the oxidative damage of the brain caused by a high-dose dexamethasone in rats (Beytut et al., 2018). Se administration prevented the decrease in glucose-6-phosphate dehydrogenase activity and GSH level in vitro (Yilmaz et al., 2006), and alleviated the reduction in antioxidant enzyme activities and GSH level in vivo caused by prednisolone (Beytut and Aksakal, 2003). Se may inhibit GR hormone binding activity (An et al., 2023). It is also possible that Se works by directly increasing antioxidant capacity and eliminating ROS which was induced by, but not limited to cortisol and LPS (Torres et al., 2021; Zakeri et al., 2021). On the other hand, the effect of cortisol on Se antioxidation was rarely reported. Despite the current result of cortisol treatment alone and the aforementioned reports that GC caused decreased selenoprotein expression in various cell types (Rock and Moos, 2009; You et al., 2009; Wray et al., 2019), we noticed that the relative abundance of *GPX1* and *GPX4* transcripts further increased by Se in the presence of cortisol and LPS (data unpublished, Supplementary Material 4). This unexpected result provided clues for further research with regard to the effect of cortisol on selenoproteins in BESC. The current result indicated that Se supplementation is capable of sustaining the global antioxidant ability of BESC, leading to decreased oxidative stress and cellular damage initiated by LPS or cortisol. Although insufficient to explain the mechanism of how cortisol further promotes the antioxidant protection of Se against BESC oxidative stress, these contents suggest the direction of further research.

## Conclusion

In primary BESC, cortisol alone induced the oxidative damage but provided an antioxidant protection in the presence of LPS. Se alleviated the LPS-induced cellular oxidative stress, which is probably achieved through activating Nrf2 pathway. At high cortisol level, Se supplement has a more significant protective effect on BESC oxidative stress. This study helps reveal the protective effect of Se against endometrial oxidative stress in cows with stress and suggests the potential regulatory mechanism.

## Supplementary Data

Supplementary data are available at *Journal of Animal Science* online.

skae260_suppl_Supplementary_Materials_1-5

## Data Availability

The data that support the findings of this study are available from the corresponding author upon reasonable request.

## Abbreviations:

ARE: antioxidant response element
BEEC: bovine endometrial epithelial cells
BESC: bovine endometrial stromal cells
COR: cortisol
CAT: catalase
GC: glucocorticoids
GPX: glutathione peroxidase
GR: glucocorticoid receptor
GSH: glutathione
HO1: heme oxygenase 1
LDH: lactate dehydrogenase
LPS: lipopolysaccharide
MDA: malondialdehyde
NQO1: NAD(P)H quinone dehydrogenase 1
NFE2L2: NFE2 like bZIP transcription factor 2
Nrf2: nuclear factor erythroid 2-related factor 2
PI3K: phosphatidylinositol-3-kinase
PKC: protein kinase C
ROS: reactive oxygen species
Se: selenium
Na_2_SeO_3_: sodium selenite
SOD: superoxide dismutase
T-AOC: total antioxidant capacity
