# Supplementary material for: Selenium suppressed the LPS-induced oxidative stress of bovine endometrial stromal cells through Nrf2 pathway with high cortisol background
Source: J Anim Sci. 2024 Sep 2;102:skae260. doi: 10.1093/jas/skae260 (PMC11445656; doi:10.1093/jas/skae260)
Supplement: skae260_suppl_Supplementary_Materials_1-5 [file skae260_suppl_supplementary_materials_1-5.docx]

**Supplementary material 1**

**Title:** Vimentin identification in primary bovine endometrial stromal cells

**Description:** The purity of stromal cell population was determined to be more than 95% by the detection of vimentin using immunocytochemistry.


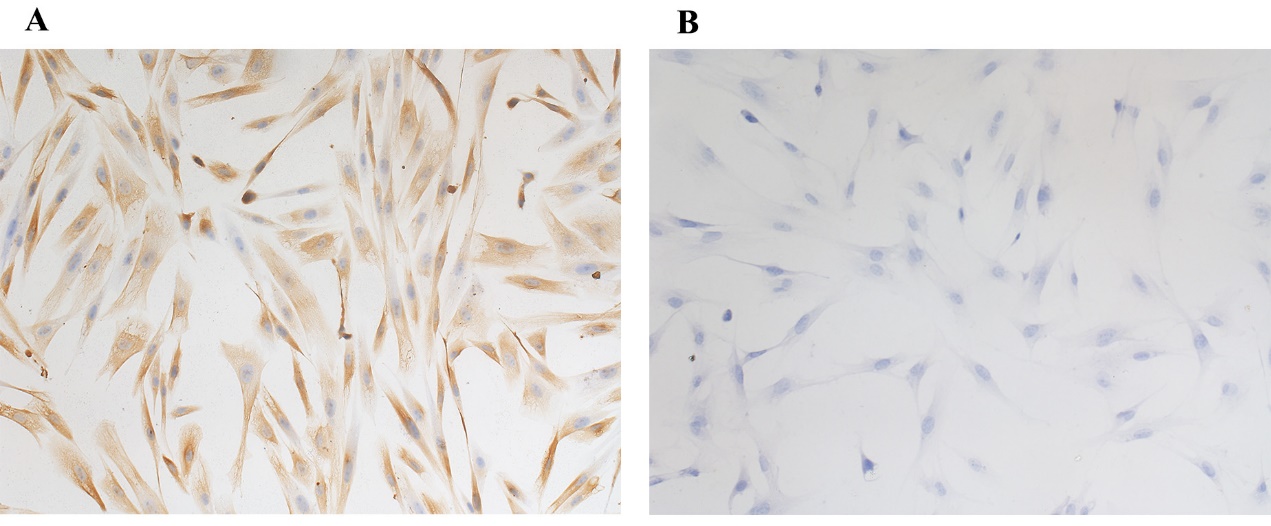


**Figure 1.** The immunocytochemistry of vimentin (A) and IgG1 (B) was presented. Scale bar represented 100 μm.

**Supplementary material 2**

**Title:** Cortisol decreased ROS and MDA levels in primary bovine endometrial epithelial cells

**Description:** Generally, cortisol resulted in a decrease (*p* < 0.05) of ROS and MDA levels in bovine endometrial epithelial cells, with the lowest level (~ 0.6-fold for ROS, ~0.9-fold for MDA) observed in 15 ng/mL cortisol intervention group.


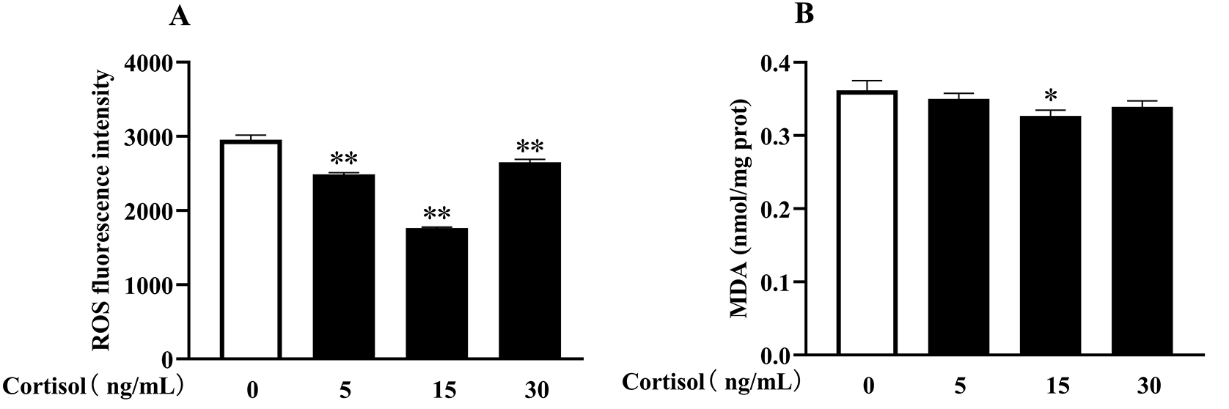


**Figure 2**. Cortisol decreased ROS and MDA levels in primary bovine endometrial epithelial cells. The cells were stimulated with 5, 15, and 30 ng/mL cortisol for 12 h to detect the changes in ROS (A) and MDA (B). COR, cortisol. MDA, malondialdehyde. ROS, reactive oxygen species. The data were presented as means ± SEM (n = 3). **p* < 0.05, ***p* < 0.01, versus the control group.

**Supplementary material 3**

**Title:** The effect of cortisol on the oxidative stress in primary bovine endometrial epithelial cells

**Description**: In general, LPS of 1 μg/mL induced (*p* < 0.01) the production of ROS and MDA. Compared with the LPS group, no change (*p* > 0.05) was found in the levels of ROS and MDA in the cells co-treated with 5 ng/mL cortisol and LPS. Cortisol of 15 and 30 ng/mL mildly reduced (*p* < 0.01) the levels of ROS and MDA in the cells with oxidative stress.


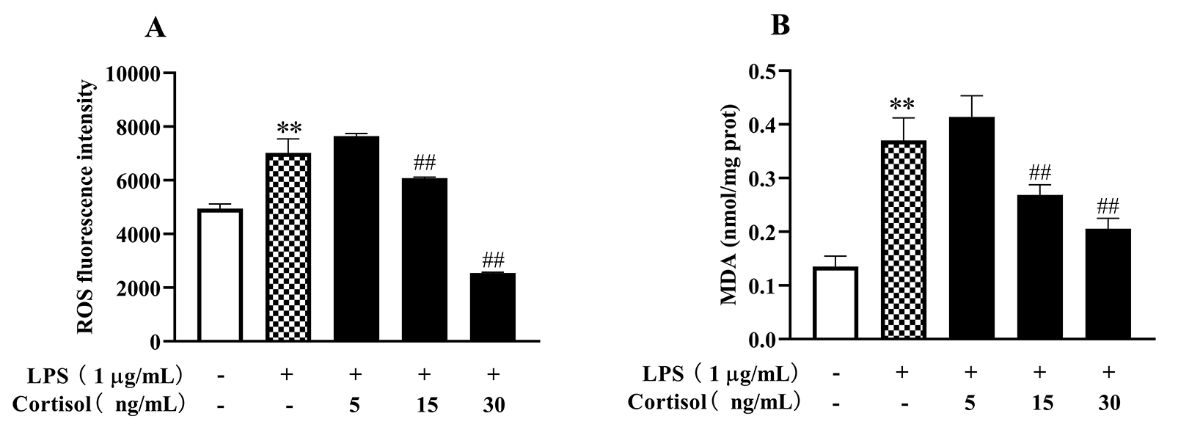


**Figure 3**. The effect of cortisol (5, 15 and 30 ng/mL) on oxidative injury of primary bovine endometrial epithelial cells. The oxidative stress model was established by LPS (1 μg/mL) stimulation. The cells were co-treated with cortisol and LPS for 12 h to detect the changes in ROS (A) and MDA (B). COR, cortisol. LPS, lipopolysaccharide. MDA, malondialdehyde. ROS, reactive oxygen species. The data were presented as the means ± SEM (n = 3). **p* < 0.05, ***p* < 0.01, versus the control group. ^#^*p* < 0.05, ^##^*p* < 0.01, versus the LPS group.

**Supplementary material 4**

**Title:** Selenium increased the relative abundance of *GPX1* and *GPX4* transcripts

**Description:** Since Se acts as selenoproteins, we examined the effect of selenium on the relative abundance of *GPX1* and *GPX4* transcripts. As a result, Se supplementation led to a greater (*p* < 0.05) abundance of *GPX1* and *GPX4* transcripts with or without the presence of LPS, and the increment was more pronounced at 24 h (5- to 8-fold) than that at 12 h (around 2-fold). The increased amplitude of *GPX* transcripts is more obvious (*p* < 0.01) in cells treated with 4 μM Se than those treated with 2 μM Se. The presence of LPS seemed to reduce (*p* < 0.05) the abundance of *GPX4* transcripts because of the difference between the LPS and LPS-Se groups, and between the Se-COR and the LPS-Se-COR groups. It seemed to suggest that LPS negatively impact *GPX4* expression. Surprisingly, the abundance of *GPX1* and *GPX4* transcripts was even greater (*p* < 0.05) in Se-COR group than that in Se group, and in the group of LPS-COR-Se than that in the LPS-Se group.


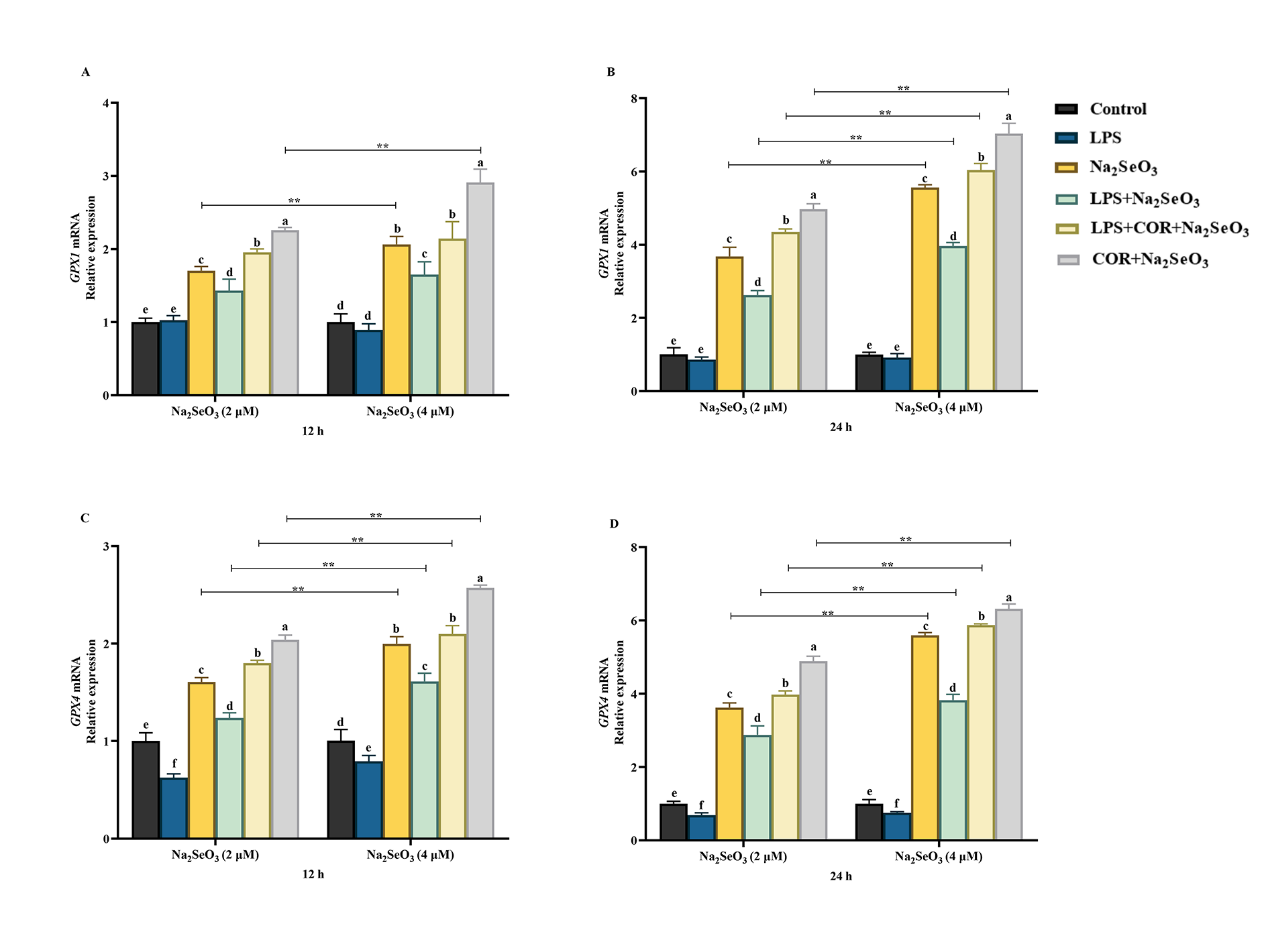


**Figure 4**. The effect of selenium on the relative abundance of *GPX1* (A, B) and *GPX4* (C, D) transcripts in primary bovine endometrial stromal cells. The cells were pretreated with 2 or 4 μM sodium selenite (Na_2_SeO_3_) for 12 h, followed by the treatment of lipopolysaccharide (LPS) and/or cortisol (COR). After treatment for 12 (A, C) or 24 h (B, D), the cells were collected for RNA extraction and quantitative PCR detection. *GPX*, glutathione peroxidase. The data were presented as means ± SEM (n = 3). Different letters indicate statistically significant differences (*p* < 0.05) among the treatment groups. The asterisks showed significant difference between the two Se concentrations within the same treatment group (***p* < 0.01).

**Supplementary material 5**

**Title:** The effects of cortisol on the oxidative stress in bovine endometrial epithelial cells

**Description:** LPS caused cellular oxidative stress, which was alleviated by the addition of cortisol. Compared with the LPS-COR group, the supplementation with Na_2_SeO_3_ reduced (*p* < 0.01) the ROS level (~0.7-fold). The level of MDA tended to be lower in LPS-COR-Se groups than that in the LPS-COR group, but without statistical significance (*p* > 0.05). On the basis of LPS and COR treatment, the presence of Se further promoted the antioxidant enzyme activities, which seemed to be dose-dependent. Specifically, the level of T-AOC, CAT, and SOD did not change (*p* > 0.05) in response to 1 μM Na_2_SeO_3_, but increased (*p* < 0.01) after 2 and 4 μM Na_2_SeO_3_ treatment (~1.1- to 1.2-fold). The protein content of GPX (~4-fold) was higher (*p* < 0.01) in LPS-COR-Se group than that of the LPS-COR group and showed an upward trend with the increase of Na_2_SeO_3_ concentration.


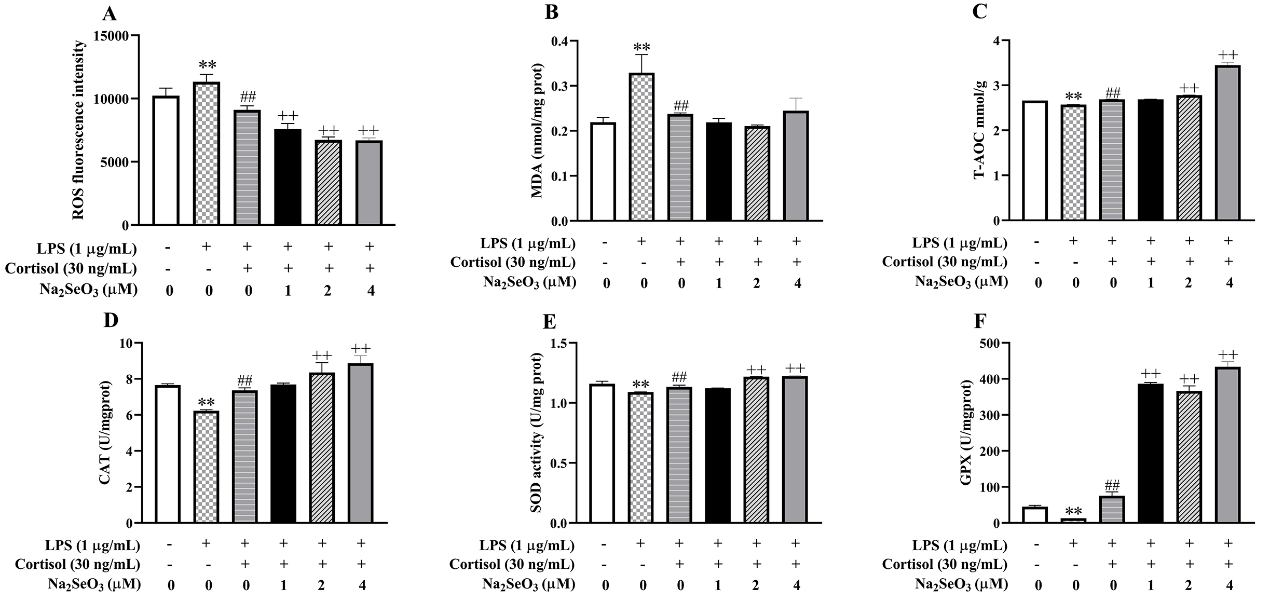


**Figure 5**. The effect of Na_2_SeO_3_ on the oxidative stress of primary bovine endometrial epithelial cells under high level of cortisol. The cells were pretreated with 1, 2, and 4μM Na_2_SeO_3_ for 12 h. Then the cells were co-treated with cortisol (30 ng/mL) and LPS (1μg/mL) for 12 h to measure the levels of ROS (A), MDA (B), and T-AOC (C), and the concentrations of CAT (D), SOD (E), and GPX (F). CAT, catalase. COR, cortisol. GPX, glutathione peroxidase. LPS, lipopolysaccharide. MDA, malondialdehyde. Na_2_SeO_3_, sodium selenite. ROS, reactive oxygen species. SOD, superoxide dismutase. T-AOC, total antioxidant capacity. The data were presented as the means ± SEM (n = 3). **p* < 0.05, ***p* < 0.01, versus the control group. ^#^*p* < 0.05, ^##^*p* < 0.01, versus the LPS group. ^+^*p* < 0.05, ^++^*p* < 0.01, versus the LPS-COR group.
